# Knowledge, attitude, and practice of stroke and thrombolysis among students preparing for undergraduate medical entrance examination in Kathmandu, Nepal

**DOI:** 10.1002/hsr2.268

**Published:** 2021-04-05

**Authors:** Ravi Ranjan Pradhan, Ashish Jha, Siddhartha Bhandari, Sujan Ojha, Ragesh Karn

**Affiliations:** ^1^ Department of Internal Medicine Provincial Hospital Janakpurdham Nepal; ^2^ Institute of Medicine Tribhuvan University Teaching Hospital Kathmandu Nepal

**Keywords:** attitude, knowledge, Nepal, practice, stroke, thrombolysis

## Abstract

**Background:**

Stroke is a major disabling disease, especially for low and middle‐income countries like Nepal. The aim of our study is to assess knowledge, attitude, and practice (KAP) among the students preparing for undergraduate medical entrance examination regarding risk factors, warning signs and symptoms, and management of stroke.

**Methods:**

A cross‐sectional, single staged study using self‐structured questionnaire intended to assess KAP about stroke and thrombolysis was conducted.

**Results:**

A total of 378 students participated in our study (53% male; mean age = 18.12 ± 0.97). Majority of the participants (88.4%) had heard about stroke. The more common risk factors identified by them were hypertension (86.2%), oily food (48%), alcohol (37.8%), and smoking (32.8%). Limb weakness, slurring of speech and facial weakness as symptoms and signs of stroke were indicated by 43.4%, 30.2%, and 18.8% of the participants, respectively. Only 23.8% of the participants had heard about thrombolysis and 10% of all could rightly mention the window period of thrombolysis. Male participants had better knowledge about smoking [86 (43.0) vs 38 (21.3); *P* < .001] and oily food [108 (54.0) vs 73 (41.0); *P* = .012] being risk factors and facial weakness [50 (37.6) vs 21 (11.8); *P* = .001] being symptom of stroke compared with females. Similarly, male participants had heard more about thrombolysis than females [68 (34.0) vs 22 (12.4); *P* < .001].

**Conclusion:**

Knowledge regarding risk factors and signs and symptoms of stroke was adequate among the students preparing for undergraduate medical entrance examination. However, knowledge about thrombolysis was poor. Male participants had better knowledge about risk factors, warning signs and symptoms of stroke, and thrombolysis compared with female.

## 1 INTRODUCTION

According to World Health Organization (WHO) Global Health Estimates in 2012, stroke was the second leading cause of death and the third leading cause of disability‐adjusted life years (DALYs).^1^ The incidence of stroke in low and middle‐income countries is in rise when compared with high‐income countries.^2^ Unfortunately, the low‐ and middle‐income countries have greater case fatality rate and a younger age of stroke onset, contributing to a high stroke burden.^2^

In 2014, WHO estimated that deaths in Nepal due to stroke were 15 333 or 9.67% of total deaths.^3^ The incidence of stroke is on the rise in Nepal; however, awareness of the regarding signs and symptoms of stroke and thrombolysis is minimal in the Nepalese populations as compared to Western populations, along with having more sophisticated health facilities.^4^ A review of the literature shows that there are several studies on the incidence, prevalence, and etiology of stroke so far but there are no any studies that could assess knowledge, attitude, and practice (KAP) toward stroke and thrombolysis in Nepal.

Stroke needs special attention in low‐ and middle‐income countries, as a lack of appropriate therapy can lead to long term morbidity and disability. Thrombolysis using alteplase is considered the drug of choice for acute ischemic stroke, it needs to be administered within 4.5 hours of onset of symptom or last known consciousness; this is referred to as the “time window” for thrombolysis.^5^ Thrombolysis, as a definitive management, has revolutionized the outcome in patients of stroke which, when done in time, greatly reduces disability associated with stroke.^6^, ^7^ Thrombolysis in Nepal has recently been started in the capital city (Kathmandu) at four tertiary care centers (as of time of writing) and so far only a handful of cases have undergone thrombolysis.^8^, ^9^ This could largely be explained by lack of knowledge regarding identification of stroke and thrombolysis among the population. Students preparing for undergraduate medical entrance examination are the prime focus of our study, as this could be an important group who could effectively inform their families and others about stroke and effective timing for thrombolysis. This study is an attempt to evaluate KAP among students preparing for undergraduate medical entrance examination regarding stroke and thrombolysis.

## 2 METHODS

### 2.1 Study design and population

This was a cross‐sectional study that was performed in a single stage at an institute in Kathmandu, Nepal from June 18, 2018 to August 17, 2018. It is a premier coaching institute for the preparation of undergraduate medical entrance examination, where students from all the 77 districts of Nepal attend for their higher education. A prior informed permission was gained from the institute for conducting the study. Students who were ≥16 years of age, preparing for undergraduate medical entrance examination and provided consent to participate were included in the study. A total of 378 students who met the inclusion criteria were selected by non‐probability method for the study. Written informed consent was obtained from all the participants.

### 2.2 Sample size determination

The sample size was calculated based on high‐risk population for stroke and these were patients with hypertension. So, prevalence of hypertension was used to calculate the sample size.

$$\text{Sample size}\;\left(n\right)=z^{2}\mathrm{pq}/L^{2}$$

where,

p = Prevalence = 44%^10^

q = 100 – p = 100 – 44= 56

L = Allowable error = 5%

Z = Variate= 1.96

Using above formula, the sample size was estimated to be 378.

### 2.3 Questionnaire

We prepared a self‐structured questionnaire in English language. The questions were formulated so as to cover basic aspects of knowledge of stroke, signs, and symptoms to recognize stroke, realize the risk factors for stroke and how to deal with someone with stroke. It also includes information about thrombolysis and extent of information the target population has regarding centers of thrombolysis. The questionnaire was pre‐tested on a sample student's population and verified. The question keys were designed to obtain single (having yes/no options and questions with only one correct response) or multiple responses. Questions 1, 2, 3, 4, 5, 6, 7 dealt with knowledge of stroke. Questions 10, 11, 12, and 13 dealt with knowledge of thrombolysis. Question 8 dealt with attitude and question 9 addressed practice related to recognition of someone with stroke. Similarly, questions 14 and 15 were framed to assess attitude regarding treatment of stroke (https://doi.org/10.6084/m9.figshare.12859529). Initially, we administered the questionnaire to 20 students for reliability testing. These students were not included in the study. Cronbach's alpha of overall scores was 0.75. Cronbach's alphas of individual question are shown in Table 1. The validated questionnaire was distributed to the participants by a group of volunteers in their classroom on 18th and 19th June 2018. Students were instructed not to discuss any questions with other participants before the completion of the study and were monitored closely.

**TABLE 1:** Cronbach's alphas of individual question (if items deleted)

S.n.	Questions	Cronbach's alpha if item deleted
1.	Have you heard about stroke?	.757
2.	Have you seen someone affected by stroke?	.732
3.	Can stroke be prevented?	.746
4.	Is stroke a communicable disease?	.741
5.	Is stroke a hereditary disease?	.738
6.	Which organ is affected in patient with stroke?	.741
7.	What are the risk factors for stroke? (Multiple options can be selected)	.716
8.	How to recognize a person with stroke? (Multiple options can be selected)	.724
9.	What should be done after recognizing a person having stroke?	.703
10.	Have you heard about thrombolysis in patients with stroke?	.737
11.	What is the appropriate time to initiate thrombolysis in patient suspected of having stroke?	.695
12.	Which of the centers provide thrombolytic therapy to stroke patients in Nepal? (Multiple options can be selected)	.716
13.	Can a person with stroke be treated?	.760
14.	Patient with stroke?	.743
15.	Which means is the best to aware people about Stroke and its management?	.737

### 2.4 Ethical considerations

This study was approved by the Institutional Review Committee (IRC) of the Institute of Medicine (IOM), Tribhuvan University Teaching Hospital, Kathmandu, Nepal on June 17, 2018. Ref no: 414 (6‐11‐E)^2^ / 074 / 075. Prior informed written consent was taken from all the participants after explaining the nature and purpose of the study.

### 2.5 Data analysis

Data were subsequently analyzed using SPSS software version 21. Results of the descriptive analysis are presented as frequency, percentage and mean ± SD. Two‐tailed Chi square test was used to analyze association between gender and KAP related to stroke and thrombolysis. A *P*‐value <.05 was considered to be statistically significant.

## 3 RESULTS

A total of 378 students participated in our study. The mean age of the participant was 18.12 ± 0.97 years and 200 (53%) among them were male. Out of 378 participants, 88.4% had heard about stroke previously and only 23.3% had seen patients who were previously diagnosed as stroke. Similarly, the majority of the participants thought that stroke is preventable (88.4%) and non‐communicable (94.7%). Stroke was thought to be hereditary disease by 159 (42.1%) participants and brain was identified as the organ involved by 172 (45.5%) participants. The four most frequent stroke risk factors identified were hypertension (86.2%), oily food (48%), alcohol (37.8%), and smoking (32.8%). Limb weakness, slurring of speech, and facial weakness were identified as symptoms and signs of stroke by 43.4%, 30.2%, and 18.8% of the participants, respectively. Male participants identified smoking [86 (43.0) vs 38 (21.3); *P* < .001] and oily food [108 (54.0) vs 73 (41.0); *P* = .012] as being risk factors and facial weakness [50 (37.6) vs 21 (11.8); *P* = .001] as being symptom of stroke more frequently when compared with female participants. However, more female participants had heard about stroke than males [168 (94.4) vs 166 (83.0); *P* = .001] [Table 2].

**TABLE 2:** General information about stroke

General information about stroke		N (%)	Gender		*P*‐value
			M	F	
Heard about Stroke?	Yes	334 (88.4)	166 (83.0)	168 (94.4)	.001
	No	44 (11.6)	34 (23.3)	10 (5.6)	
Seen someone with stroke?	Yes	88 (23.3)	54 (27.0)	34 (19.1)	.070
	No	290 (76.7)	146 (73.0)	144 (80.9)	
Can stroke be prevented?	Yes	334 (88.4)	179 (89.5)	155 (87.1)	.464
	No	44 (11.6)	21 (10.5)	23 (12.9)	
Is stroke a communicable disease?	Yes	20 (5.3)	11 (5.5)	9 (5.1)	.847
	No	358 (94.7)	189 (94.5)	169 (94.9)	
Is stroke a hereditary disease?	Yes	159 (42.1)	79 (39.5)	80 (44.9)	.285
	No	219 (57.9)	121 (60.5)	98 (55.1)	
Which organ is affected by stroke?	Brain	172 (45.5)	90 (45.0)	82 (46.1)	.835
	Others	206 (54.5)	110 (55.0)	96 (53.9)	
Risk factors?	Smoking	124 (32.8)	86 (43.0)	38 (21.3)	<.001
	Alcohol	143 (37.8)	82 (41.0)	61 (34.3)	.178
	Heredity	106 (28.0)	53 (26.5)	53 (29.8)	.479
	Oily food	181 (47.9)	108 (54.0)	73 (41.0)	.012
	Diabetes mellitus	119 (31.5)	57 (28.5)	62 (34.8)	.186
	Hypertension	326 (86.2)	169 (84.5)	157 (88.2)	.297
Signs and symptoms with positive response	Double vision	117 (31.0)	61 (30.5)	56 (31.5)	.840
	Blindness	97 (25.7)	50 (25.0)	47 (26.4)	.755
	Slurring of speech	114 (30.2)	64 (32.0)	50 (28.1)	.408
	Facial weakness	71 (18.8)	50 (37.6)	21 (11.8)	.001
	Loss of consciousness	284 (75.1)	146 (73.0)	138 (77.5)	.309
	Abnormal body movements	143 (37.8)	83 (41.5)	60 (33.7)	.119
	Weakness of limbs	164 (43.4)	71 (35.5)	93 (52.2)	.001
	Headache	185 (48.9)	99 (49.5)	86 (48.3)	.818

There were 308 (81.5%) participants who considered stroke to be a treatable disease. Patient should be taken to hospital after recognition of a stroke was selected as a response by 58.5% of the students. Only 90 (23.8%) participants had prior knowledge of the use of thrombolysis in stroke. Male participants had heard more about thrombolysis compared with females [68 (34.0) vs 22 (12.4); *P* < .001]. Out of all participants, 38 (10%) could correctly identified the window period before which definitive treatment (thrombolysis) should be started in patients with stroke. Improvement of morbidity by physiotherapy was identified by 215 (57%) participants. The majority of the participants (40.7%) was aware about of only one center in Kathmandu where thrombolysis is being performed. The majority of the participants (49.7%) thought that the best means to make people aware of stroke was community and school‐based programs (Table 3).

**TABLE 3:** Information about treatment and prognosis of stroke

Information about treatment and prognosis of stroke		N (%)	Gender		*P*‐value
			M	F	
Take patient to emergency after stroke	Yes	221 (58.5)	107 (53.5)	114 (64.0)	.275
	No	157 (41.5)	93 (46.5)	64 (36.0)	
Heard about thrombolysis?	Yes	90 (23.8)	68 (34.0)	22 (12.4)	<.001
	No	288 (76.2)	132 (66.0)	156 (87.6)	
Appropriate time to initiate definitive treatment (thrombolysis)?	Within 4.5 hours	38 (10.1)	15 (7.5)	23 (12.9)	.080
Number of centers providing thrombolysis identified	All four centers	15 (4.0)	7 (1.9)	8 (2.1)	NA
	Three centers	47 (12.4)	21 (5.6)	26 (6.9)	
	Two centers	138 (36.5)	72 (19.0)	66 (17.5)	
	One center	154 (40.7)	85 (22.5)	69 (18.3)	
	None	24 (6.3)	15 (4.0)	9 (2.4)	
Can the patient of stroke be treated?	Yes	308 (81.5)	164 (82.0)	144 (80.9)	.783
	No	70 (18.5)	36 (18.0)	34 (19.1)	
Does patient with stroke (single response is allowed)	Needs lifelong medications?	92 (24.3)	43 (21.5)	49 (27.5)	NA
	Requires family assistance throughout their life?	58 (15.3)	38 (19.0)	20 (11.2)	
	Improves with appropriate physiotherapy?	215 (56.9)	111 (55.5)	104 (58.4)	
	Is bed‐ridden for life?	13 (3.4)	8 (4.0)	5 (2.8)	
What is the best means to aware people about stroke? (single response is allowed)	Newspapers	38 (10.1)	26 (13.0)	12 (6.7)	NA
	Radio/television	62 (16.4)	37 (18.5)	25 (14.0)	
	Doctors/medical personnel	78 (20.6)	38 (19.0)	40 (22.5)	
	Posters and pamphlets	12 (3.2)	6 (3.0)	6 (3.4)	
	Community and school based programs	188 (49.7)	93 (46.5)	95 (53.4)	

## 4 DISCUSSION

The students participated in our study belong to a particular institute in an urban setting, who have adequate general information on stroke. Only about one‐tenth of them had not heard about stroke previously. However, we discovered that less than a quarter of them had seen patient who was previously diagnosed as stroke, which can be attributed to their limited information about symptoms of stroke. Less than half of the students could correctly identify the organ affected in stroke, which was significantly higher than shown by the study of Kaddumukasa et al^11^ conducted in the general population in Uganda.

Knowledge about smoking and oily food being risk factors and facial weakness being symptom of stroke was found to be more in male respondents compared with female. This is in contrast with study of Rachmawati et al,^12^ who found that gender had no significant influence in determining knowledge about stroke. This finding may be due to greater accessibility of male respondents to social media and the internet in Nepal. Since our study included only students preparing for undergraduate medical entrance examination, the age difference among the participants was not sufficient to correlate with level of knowledge. We found that hypertension was the most commonly identified risk factor for stroke (86.2% of the participants), which was similar to the studies by Haghighi et al^13^ and Yadav et al,^14^ conducted in populations of developing countries. Less than half of them could mention diabetes mellitus, oily food, and alcohol intake as risk factors for stroke. Only one‐third of the participants could identify smoking as a risk factor for stroke, which alarms the lack of awareness among youth (Table 1). This finding is in contrast with study of Haghighi et al,^13^ in which 87.8% of the respondents could identify smoking as a risk factor of stroke. In our study, the symptoms most commonly identified were loss of consciousness, headache, and weakness of limbs which is consistent with findings of Kaddumukasa et al^11^ in rural and urban population. About one‐third of the respondents identified slurring of speech as one of the important symptoms of stroke. Similar finding was observed in the study from Saudi Arabia conducted by Alreshidi et al.^15^ Facial deviation, which is an important sign was recognized by only 18.8% of the study population and this finding is in accordance to study performed by Alreshidi et al.^15^

Approximately, 60% of the students opted to take patients to hospital after witnessing a suspected case of stroke, which is not satisfactory when considered in the background of urban setting and higher level of education among our study population. A review article by Sowtali et al^16^ discovered that 31% to 73% people in developed nations have awareness to dial the emergency system and take the patient to hospital after witnessing a case of suspected stroke. Functional deterioration or disability may occur if there is any delay in seeking emergency treatment. Less than a quarter of the participants knew about thrombolysis, and only 10% could correctly identify the window period for thrombolysis. Knowledge regarding the window period for thrombolysis among the general population is important because it significantly affects the onset of stroke to reaching hospital time, and thereby preventing the loss of time in the golden hours.^17^, ^18^ The students had the least information about the centers providing thrombolysis service as only 4% of them could rightly respond all the four centers where thrombolysis is being routinely performed. Knowledge of appropriate centers that can provide thrombolysis and their locations has been found to significantly affect pre‐hospital delay after the relatives of the victim are able to identify stroke.^19^, ^20^

A good number of students (56.9%) were of the opinion that patients with stroke can improve with appropriate physiotherapy. Community and school‐based programs and health education provided by doctors and medical personnel were considered to be the most effective methods to make people aware of stroke.

Low‐middle income countries like Nepal should largely rely upon preventive measures and awareness campaigns to reduce occurrence of stroke, and morbidity and mortality related to stroke, especially when advanced treatment strategies like endovascular thrombectomy is far from being established in the country.^21^, ^22^ A high level of political and inter‐organizational commitment needs to be established to make high school students aware of the risk factors of stroke, identifying a victim of stroke and thereby improving their response to minimize loss of time before reaching the appropriate health care center.

Being a questionnaire‐based cross‐sectional study, this survey has its own limitations. The sample size of our study is small. Since this study is conducted among high‐school science students, the level of knowledge can be expected to be more as compared to the general population. Hence, this does not provide a real projection of knowledge about stroke and thrombolysis in the community. As the study was conducted in the capital city (Kathmandu), the status of baseline knowledge among high school students in other parts of the country might be less than that found in ours, as the facilities regarding definitive stroke management (ie, thrombolysis) are limited beyond the capital. Despite lack of awareness and lack of facility, unavailability of transportation and challenging geographical status of a Himalayan country have a huge impact in increasing pre‐hospital delay which could not be explored by this study.

## 5 CONCLUSION

Study on KAP is essential to increase the awareness of risk factors, warning signs and symptoms, thrombolysis and secondary prevention in patients with stroke. Dissemination of information among the general population on stroke and the golden hour of thrombolysis is vital to reduce stroke‐related morbidity and mortality in developing nations like Nepal. Having found that, through ensuring sufficient knowledge of stroke among students preparing for undergraduate medical entrance examination, it is possible to promote the level of KAP among general population toward stroke and thrombolysis as these students could effectively aware their family members and surroundings about stroke and thrombolysis. We recommend further large‐scale studies should be carried among Nepalese people about stroke and thrombolysis to reduce delay of patients reaching the hospital after they get stroke or seen someone with stroke.

## CONFLICT OF INTEREST

Authors declare no any conflict of interest.

## AUTHOR'S CONTRIBUTION

Conceptualization: Ravi Ranjan Pradhan, Ashish Jha, Ragesh Karn.

Formal Analysis: Ravi Ranjan Pradhan, Ashish Jha.

Writing ‐ Original Draft Preparation: Ravi Ranjan Pradhan, Ashish Jha, Siddhartha Bhandari, Sujan Ojha, Ragesh Karn.

Writing ‐ Review & Editing: Ravi Ranjan Pradhan, Ashish Jha, Siddhartha Bhandari, Sujan Ojha.

## TRANSPARENCY STATEMENT

Authors confirm that manuscript is an honest, accurate, and transparent account of the study being reported and no important aspects of the study have been omitted.

## Data Availability

*Underlying data* Figshare: Knowledge, Attitude, and Practice of Stroke and Thrombolysis among Students Preparing for Undergraduate Medical Entrance Examination in Kathmandu, Nepal. https://doi.org/10.6084/m9.figshare.12859895 The project contains the following underlying data: Data Stroke.sav (SPSS data entry sheet of all the participants). *Extended data* Figshare: Knowledge, attitude, and practice of stroke and thrombolysis among students preparing for undergraduate medical entrance examination in Kathmandu, Nepal. https://doi.org/10.6084/m9.figshare.12859478 The project contains the following underlying data: Validity.sav (SPSS data entry sheet for validity testing). Figshare: Knowledge, attitude, and practice of stroke and thrombolysis among students preparing for undergraduate medical entrance examination in Kathmandu, Nepal. https://doi.org/10.6084/m9.figshare.12859529 The project contains the following underlying data: Questionnaire IRB ‐ Copy.docx (Validated questionnaire).
